# Aggressive Male Breast Cancer—Clinical and Therapeutic Aspects Correlated with the Histopathological Examination: A Case Report and Literature Review

**DOI:** 10.3390/medicina59122167

**Published:** 2023-12-14

**Authors:** Ana-Maria Petrescu, Nicolae-Daniel Pirici, Anca-Ileana Ruxanda, Liviu Vasile, Mircea Pîrșcoveanu, Ștefan Paitici, Gabriel-Sebastian Petrescu, Alexandru Claudiu Munteanu, Ramona-Andreea Matei, Daniel Dumitrache, Andreas Donoiu, Stelian-Ștefăniță Mogoantă

**Affiliations:** 1 Doctoral School, University of Medicine and Pharmacy of Craiova, 200349 Craiova, Romania; ana.petrescu95@gmail.com (A.-M.P.); angimatei@yahoo.com (R.-A.M.); 2 3rd General Surgery Clinic, Emergency County Hospital, 200642 Craiova, Romania; anca.ruxanda@umfcv.ro (A.-I.R.); liviu.vasile@umfcv.ro (L.V.); mircea.pirscoveanu@umfcv.ro (M.P.); alexandru.munteanu@umfcv.ro (A.C.M.); dumitracheddaniel@yahoo.com (D.D.); stelian.mogoanta@umfcv.ro (S.-Ș.M.); 3 Department of Research Methodology, University of Medicine and Pharmacy of Craiova, 200349 Craiova, Romania; daniel.pirici@umfcv.ro; 4 Department of Surgery, University of Medicine and Pharmacy of Craiova, 200349 Craiova, Romania; 5 Department of Oral and Maxillofacial Surgery, University of Medicine and Pharmacy of Craiova, 200349 Craiova, Romania; gabriel.petrescu@umfcv.ro

**Keywords:** male breast cancer, histopathological examination, immunohistochemical reactions, treatment

## Abstract

Breast cancer is often seen as a disease that occurs in women, but it can also appear in men in a very small percentage, below 1%. Men have a minimal amount of breast tissue compared to women, which has the potential to become malignant in a similar way to women, although much less frequently. A patient presented with advanced local invasion due to the low amount of breast tissue, with the tumor quickly invading the adjacent structures. Histopathological and immunohistochemical examinations have an extremely important role in the pathology of breast cancer. Given that male breast cancer is rare and there are not enough surgeons specializing in breast surgery in our country, there is a lack of experience in the management and early diagnosis of this type of cancer, which will be highlighted in this article.

## 1. Introduction

Male breast cancer (MBC) is a rare disease, representing less than 1% of all breast cancers worldwide and approximately 1% of cancers that occur in men [1,2].

Because MBC is rare, not much is known about the disease, and treatment recommendations are typically extrapolated from data available from clinical trials enrolling female breast cancer (BC) patients [3].

The risk factors are also different for men compared to women. Most affected men do not have associated risk factors. However, some hormonal, genetic and environmental factors have been involved in pathogenesis [4]. MBC is more likely to occur in the case of a BRCA2 mutation, unlike female BC, which is more likely to occur in the case of a BRCA1 mutation [5]. Also, another known risk factor for men is low androgenic status; respectively, alteration of hormonal balance with excessive stimulation of estrogen was associated with an increased risk of BC [6,7]. This may occur in some testicular abnormalities (undescended testis, orchitis), congenital inguinal hernia, infertility, or orchiectomy [8,9].

The risk of MBC increases with age, compared to women whose rate depends on the usual age of menopause; this supports the hypothesis that the midlife change in the rate of increase with age in women is due to the reduction in ovarian hormone production at menopause [10].

Invasive carcinoma of no special type (NST) is the most common MBC [11]. Histologic types of ductal origin occur relatively more frequently in men than in women, reflecting the absence of lobular structures in the normal male breast; those of lobular origin are very uncommon in men [10,12].

Although significant efforts have been made to increase awareness of female BC for screening, diagnosis and treatment options, research into MBC has been limited. The management in terms of investigations and treatment options for MBC has been mainly based on the adoption of practices developed to treat female patients with BC [13].

## 2. Case Presentation

A 72-year-old male, with no significant familial history, noticed the presence of a tumor in his right breast 1 year ago; initially painless, it became bigger within 6 months and painful with erythematous peritumoral skin. He sought medical attention at a private clinic in January 2022, where they performed an incisional biopsy of the right breast tumor, with the histopathological (HP) examination showing an extended area of invasive lobular carcinoma to the edges of the fragment with areas of ductal carcinoma of low grade in situ and on areas of infiltrative breast carcinoma NST and immunohistochemical (IHC) reactions, indicating invasive breast carcinoma, NST, positive CK7 uniformly in the tumor arrangements, negative CK20, positive mammaglobin in over 80% of tumor cells, positive E-cadherin uniformly, positive estrogen receptor (ER), positive progesterone receptor (PR), Ki67: 56%, and negative HER2.

**Figure 1:**
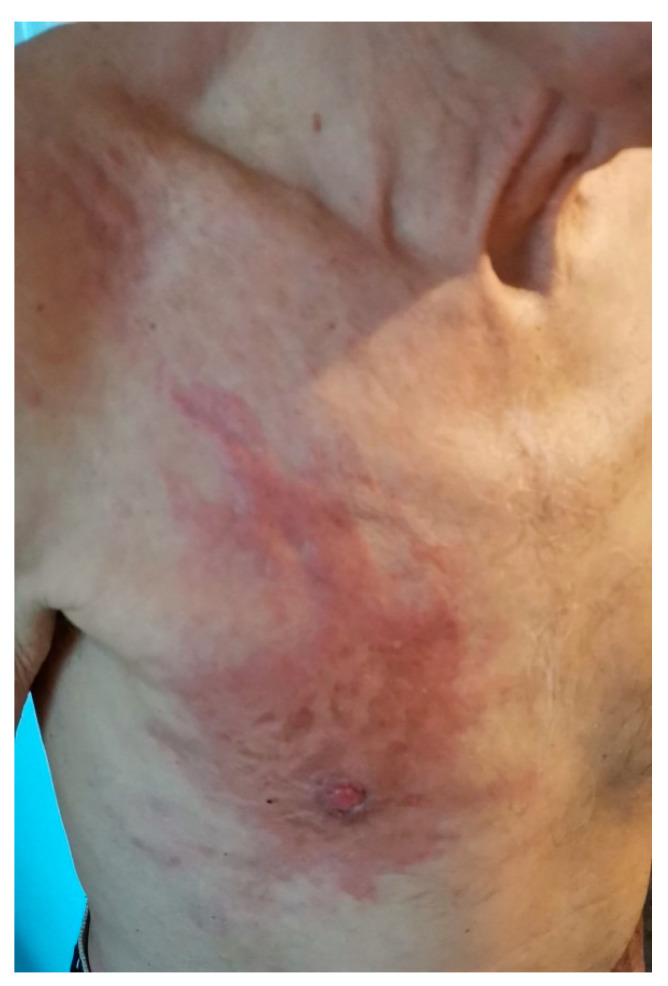
Appearance before starting neoadjuvant chemotherapy.

The patient received four cycles of neoadjuvant epirubicin + cyclophosphamide and four cycles of docetaxel (Figure 1) between March and September 2022 and was hospitalized in the 3rd Surgery Clinic, Emergency County Hospital, Craiova, Romania, in September 2022 for the presence of a tumor in the central quadrant of the right breast, with the presence of local pain, nipple retraction and erythematous periareolar skin. The patient had a partial response to chemotherapy, which is why he was proposed for surgical removal.

**Figure 2:**
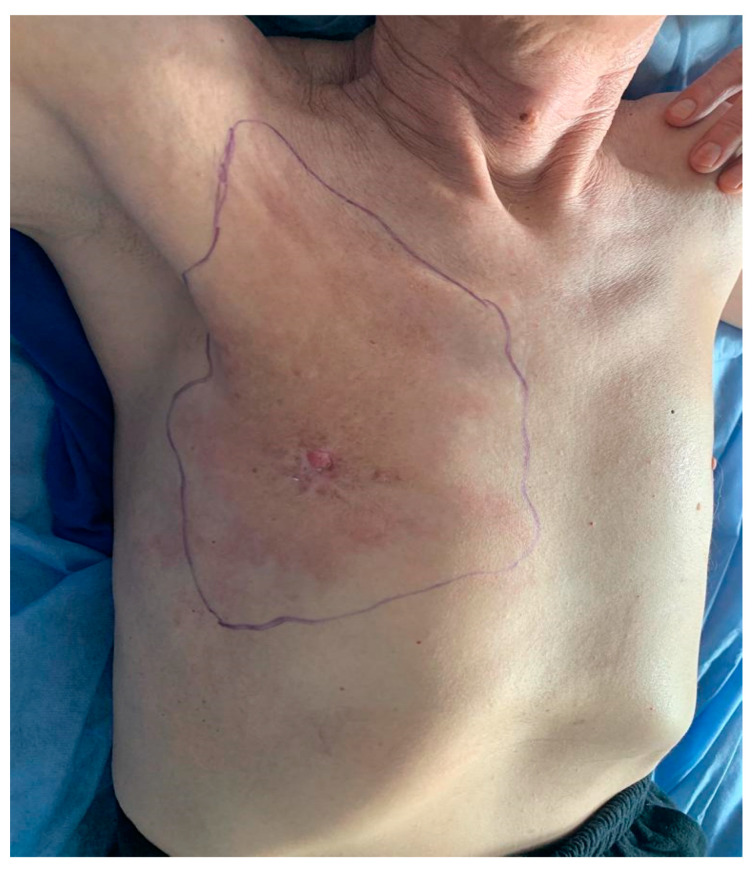
Preoperative image of the patient’s right breast after chemotherapy, with the presence of nipple retraction and erythematous periareolar skin.

The inspection revealed that the right mammary gland was found to be slightly enlarged, and the right prepectoral area was tumoral infiltrated of approximately 15/12 cm, with brown overlying skin, without pathological secretions (Figure 2). On palpation, at the level of the right mammary gland, the area of induration can be detected at the level of the central and supero-external quadrants, with the presence of an obvious tumor mass in the central quadrant, increased consistency, adhesion to the deep and superficial planes and without pathological discharge when the nipple is expressed. The right axilla had multiple lymph nodes with diameters between 1 and 2 cm, hard and adherent to the deep and superficial planes. The left breast was found clinically normal. The left axilla with multiple lymph nodes had diameters between 1 and 2 cm, mobile on the deep and superficial planes. Supraclavicular adenopathies were not detected.

Ultrasound (US) of the right breast revealed skin with lymphatic edema, thickened by 0.5 cm, with a retracted muriform nipple; heterogeneous glandular tissue, difficult to delimit from the superficial muscular fascia; a polynodular structure and numerous confluent hypoechoic nodules with a diameter between 0.23 and 0.56 cm, located predominantly in the central and supero-external quadrants. In the right axilla region, hypoechoic nodules were found and were well delimited and vascularized, with sizes between 1.38 cm and 2.1 cm; in the left axilla, multiple nodules were also found between 0.88 and 2.27 cm, with the same characteristics.

A computed tomography (CT) scan of the chest, abdomen and pelvis revealed, at the level of the right mammary gland, a tissue mass of 30/7 mm, centrally located and tangential to a subcutaneous thickening with a maximum thickness of 4.5 mm, nonspecific infracentimetric nodes in the lower right paratracheal of 13/9 mm, para-aortic adenopathy with a maximum size of 9/7 mm, and a lower left paratracheal node of 9/6 mm. Without the imagistic appearance of bone, lung or liver metastases.

The usual blood tests collected (complete blood count, coagulation profile, urea, creatinine, transaminases, bilirubin test, electrolyte test and tumor markers) were within normal limits, with the exception of the tumor marker CA 15-3, which had a value of 56.01 U/mL.

**Figure 3:**
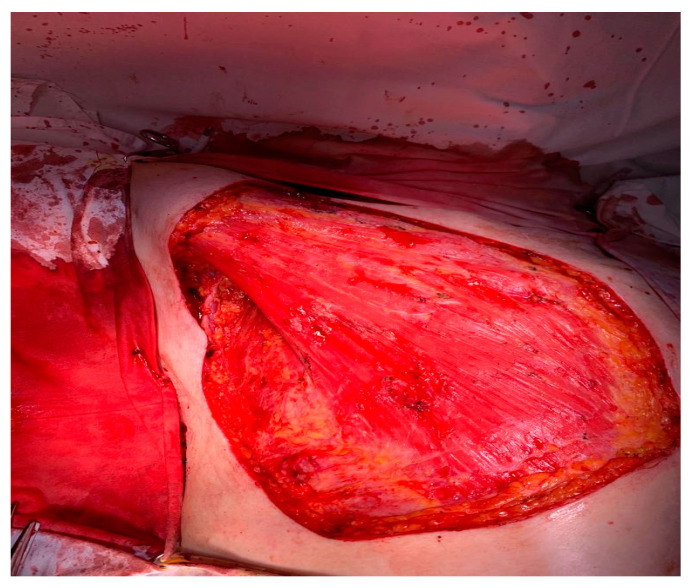
Image of pectoralis major muscle after its partial resection and right mastectomy.

**Figure 4:**
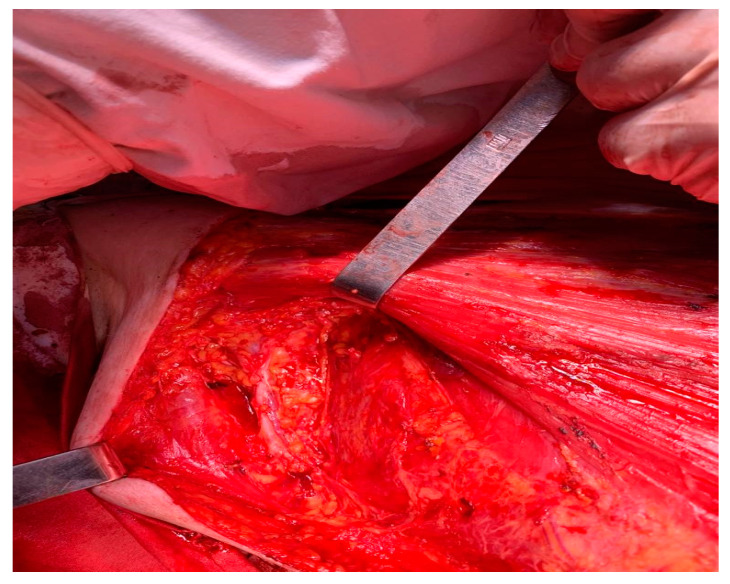
Image of the axilla after lymphadenectomy level I, II and level III axillary sampling.

**Figure 5:**
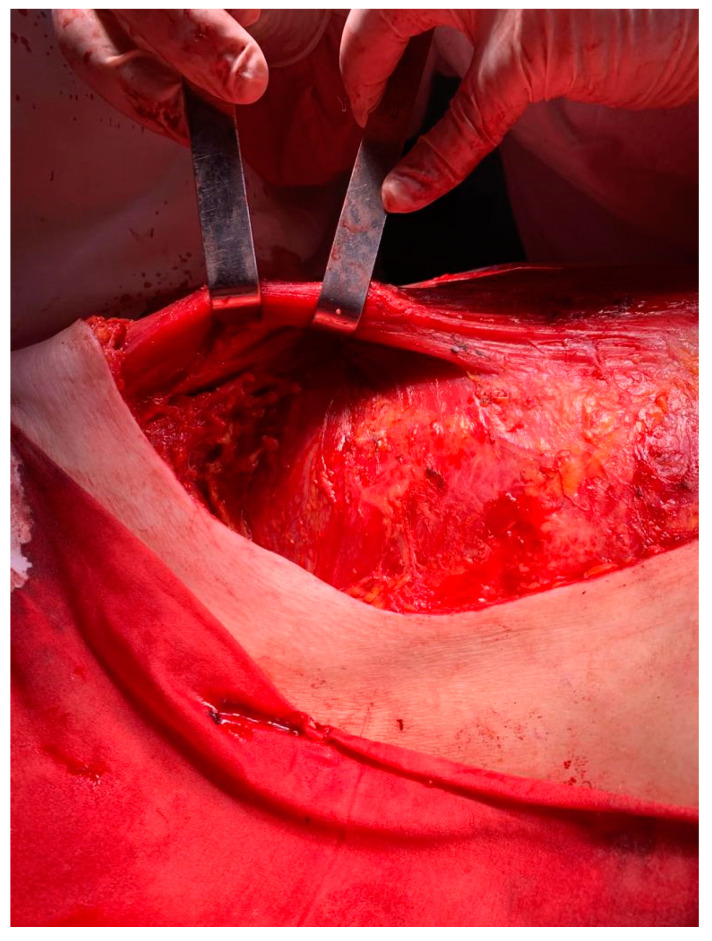
Image of retropectoral dissection and multiple adenopathic blocks that encased the axillary vein.

**Figure 6:**
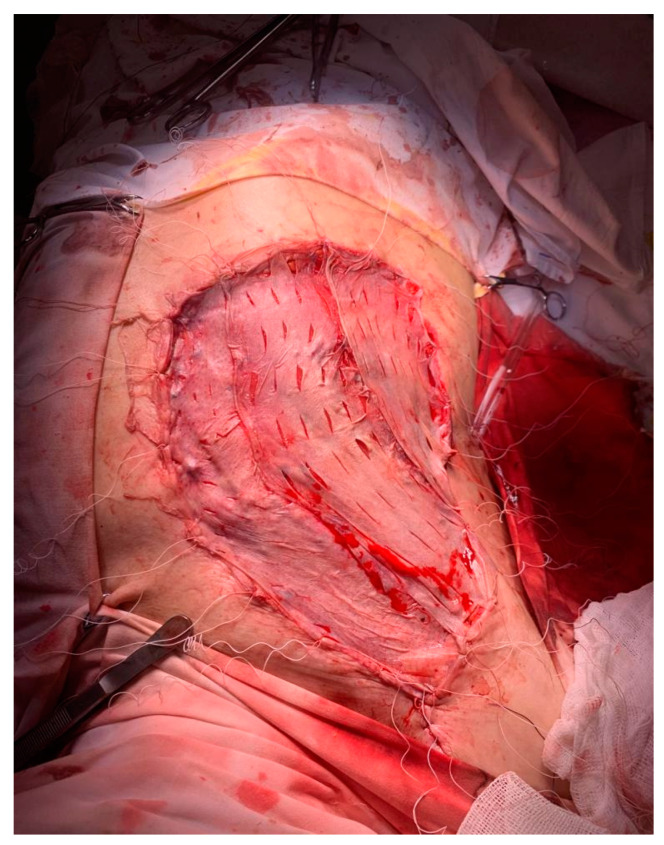
Image of the three split skins used for closure of skin’s defect.

After the usual preoperatory preparation, surgery was performed, and in the subareolar region, the apparent invasion of the pectoralis major muscle was found, and its partial resection was performed (Figure 3). There were also multiple adenopathic blocks that encased the axillary vein. The dissection of the axillary nodes was difficult due to adhesions to the axillary vein (Figure 4). The lesion evaluation imposed the performance of a right mastectomy with level I and II axillary lymphadenectomy and level III axillary sampling (Figure 4 and Figure 5). Three split skin flaps harvested from the thighs bilaterally were used for the closure of the skin’s defect in the same operation (Figure 6).

**Figure 7:**
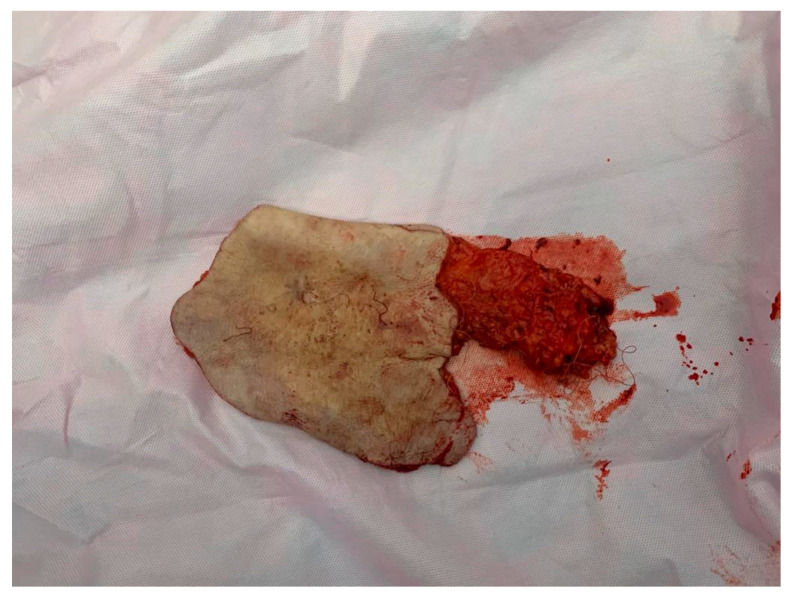
Image of the mastectomy piece, covered by skin, with the presence of nipple retraction and axillary adipose tissue.

**Figure 8:**
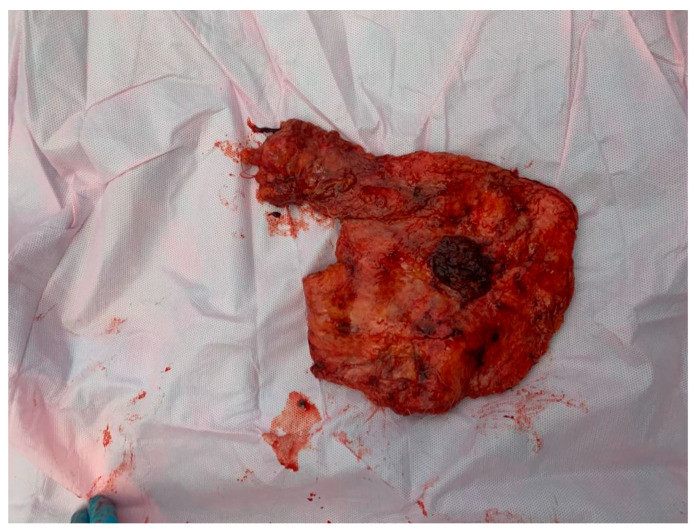
The posterior face of the resection piece with the presence of a 3 cm pill of the pectoralis major muscle.

For establishing the HP diagnosis, the specimen (Figure 7 and Figure 8) was sent to the Laboratory of Pathological Anatomy.

The HP examination of the mastectomy piece—fixed in formalin, included in paraffin and stained with Hematoxylin–Eosin (HE)—revealed infiltrative breast carcinoma with the most likely infiltrative micropapillary pattern, with areas of tumor necrosis, perineural invasion and numerous tumor emboli present. The resection limits were uninvaded, and all 12 resected lymph nodes showed carcinoma metastasis. The pathological staging was T2N3a (stage IIIC).

Due to his advanced age and anesthesia and surgery risks, the patient refused to perform a mediastinoscopy and core biopsy from the left axilla.

For a better characterization, IHC investigations were performed using the following antibodies: anti-Ki67 (monoclonal mouse anti-human Ki67, clone MM1, no dilution, Leica Bond), anti-CD34 (monoclonal mouse anti-human CD34, clone QBEnd/10, no dilution, Leica Bond), anti-ER (monoclonal mouse anti-human ER, clone 6F11, no dilution, Leica Bond), anti-PR (monoclonal mouse anti-human PR, clone 16, no dilution, Leica Bond), anti-E-cadherin (monoclonal mouse anti-human E-cadherin, clone 36B5, no dilution, Leica Bond), anti-EMA (monoclonal mouse anti-human EMA, clone GP1.4, no dilution, Leica Bond) and HER2 (monoclonal mouse anti-human c-erbB-2, clone CB11, 1:40 dilution, Novocastra).

**Figure 9:**
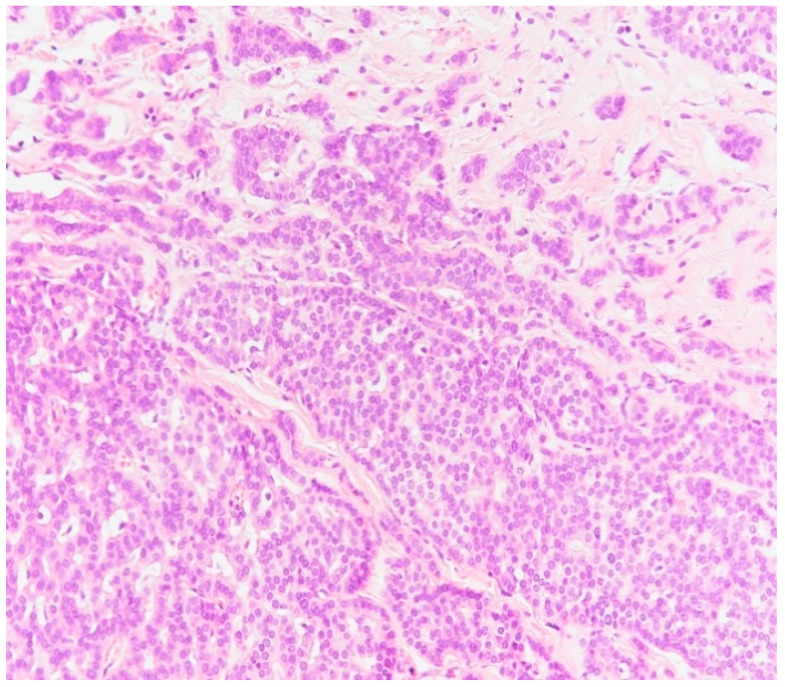
Image of a general solid pattern of the tumor with some infiltrative elements (HE staining, ×20).

**Figure 10:**
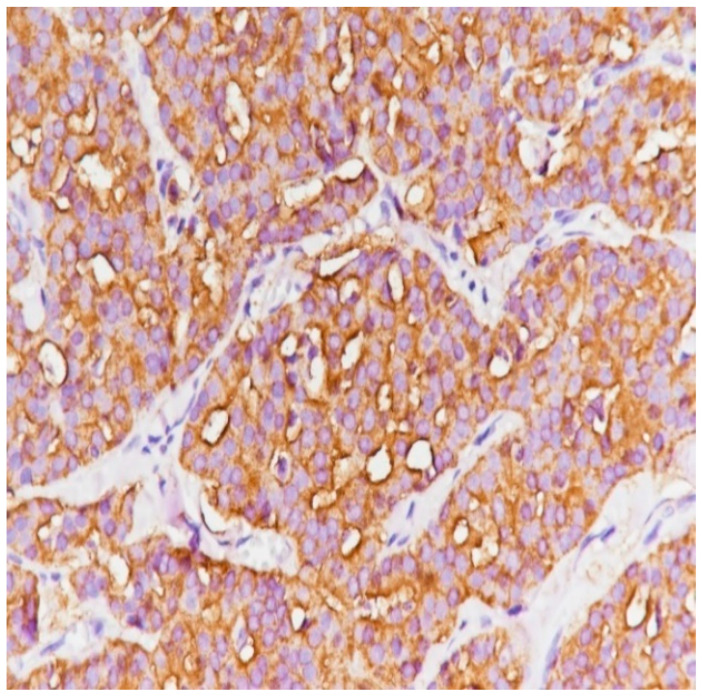
Epithelial membrane antigen (EMA) with an intense apical expression (immunolabeling with anti-EMA antibody, ×40).

**Figure 11:**
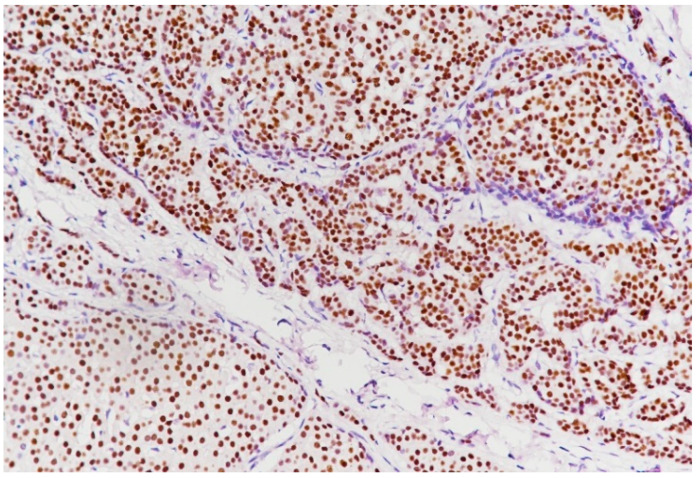
ER intensely positive (immunolabeling with anti-ER antibody, ×20).

**Figure 12:**
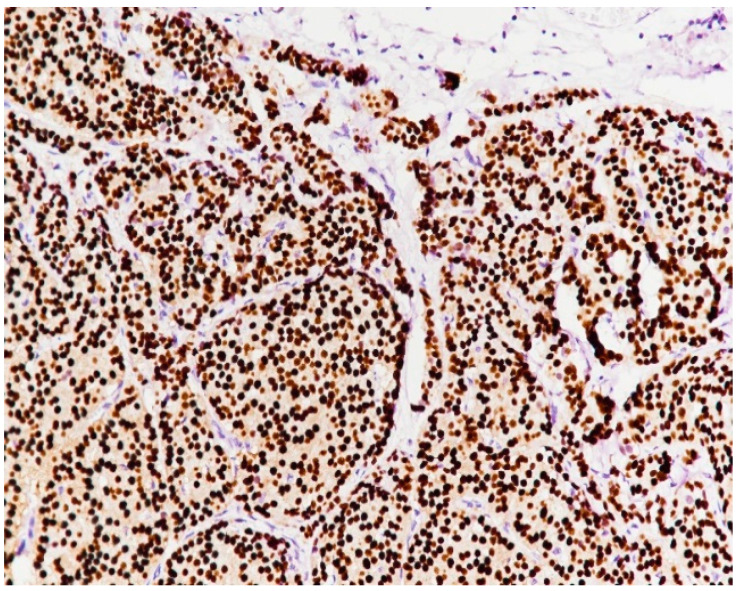
PR intensely positive (immunolabeling with anti-PR antibody, ×20).

**Figure 13:**
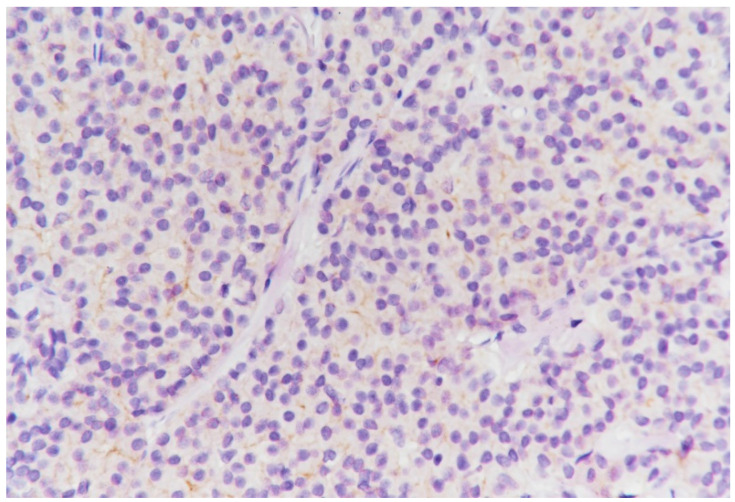
Very rare membrane HER2 elements, score 0 (immunolabeling with anti-c-erbB-2 antibody, ×40).

**Figure 14:**
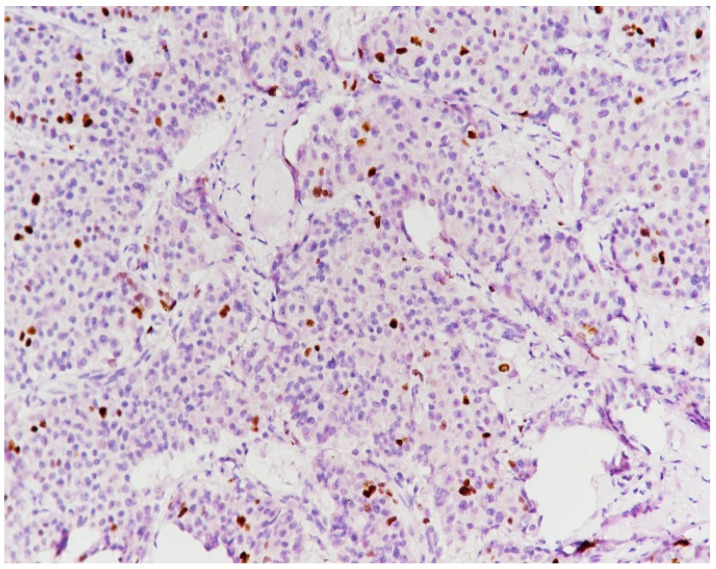
Tumor cells with low IHC reaction to anti-Ki67 antibody (immunolabeling with anti-Ki67 antibody, ×20).

**Figure 15:**
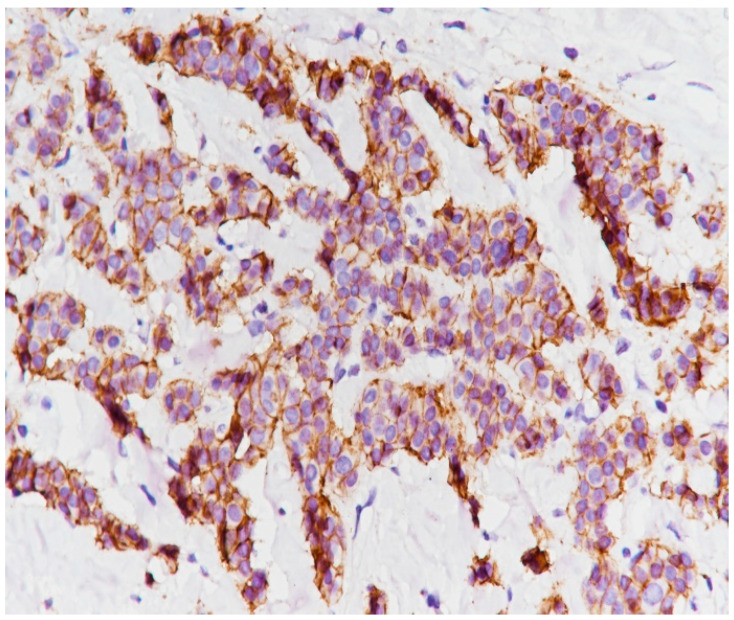
E-cadherin expression maintained in the infiltrative areas (immunolabeling with anti-E-cadherin antibody, ×20).

**Figure 16:**
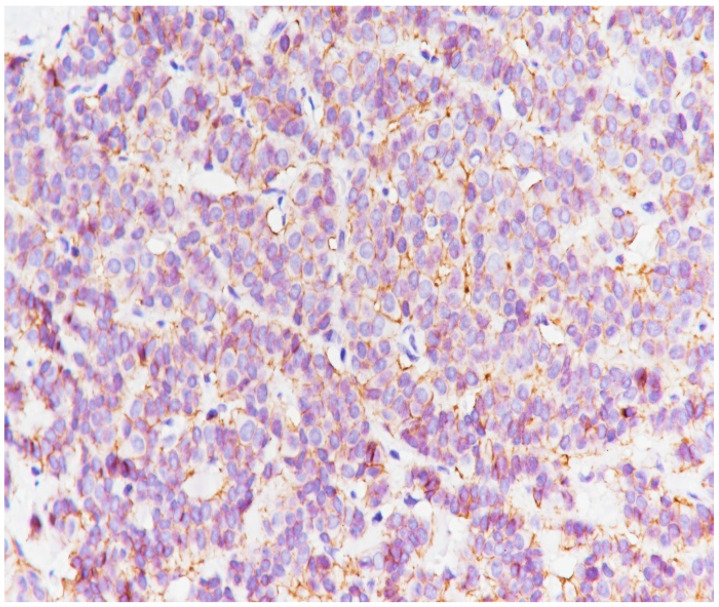
E-cadherin expression decreased in a solid area (immunolabeling with anti-E-cadherin antibody, ×20).

**Figure 17:**
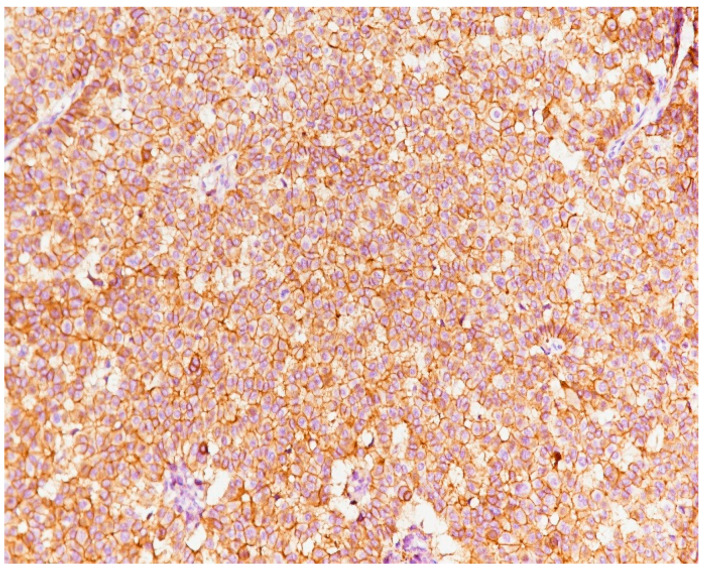
E-cadherin expression preserved in another solid area (immunolabeling with anti-E-cadherin antibody, ×20).

**Figure 18:**
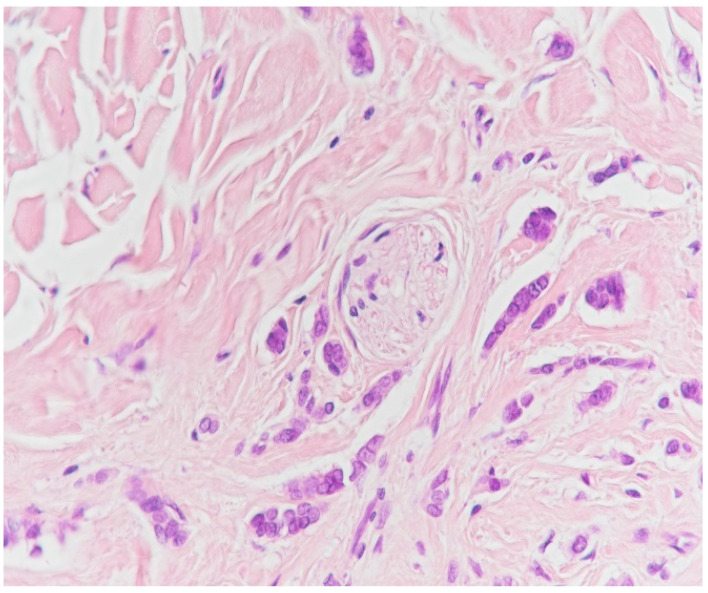
Neural invasion (HE staining, ×40).

**Figure 19:**
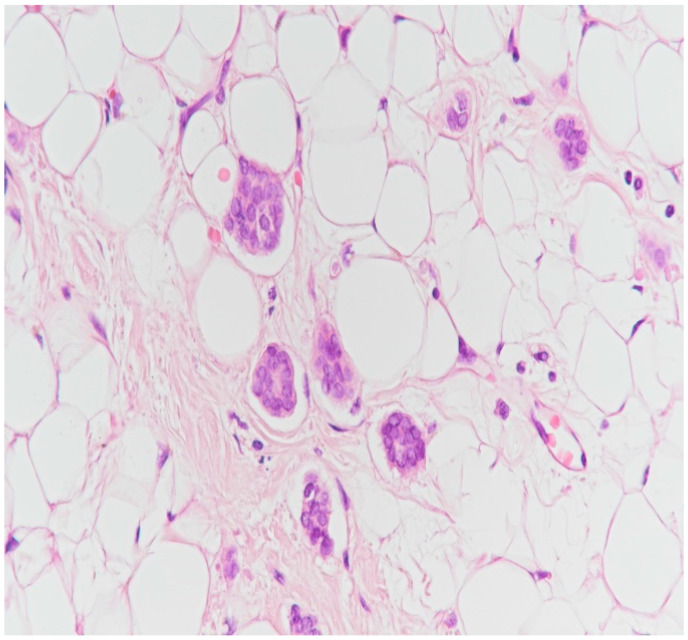
Suspicion of emboli in the vessels before immunohistochemistry for CD34 (HE staining, ×40).

**Figure 20:**
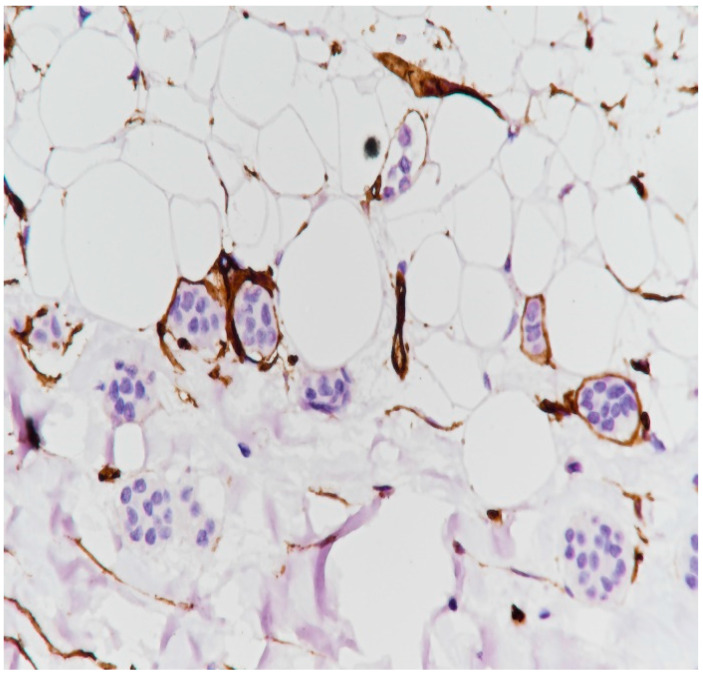
The presence of emboli at the level of small vessels (immunolabeling with anti-CD34 antibody, ×20).

The IHC study showed that the tumor had a general solid pattern with infiltrative elements (Figure 9) and intense apical expression for epithelial membrane antigen (EMA) (Figure 10) and was ER-positive (Figure 11), PR-positive (Figure 12) and HER2-negative (Figure 13). The Ki-67 cellular proliferation index was low, with about 10% of the tumor cells (Figure 14). E-cadherin expression was studied in infiltrative areas as well as in solid areas of the tumor (Figure 15, Figure 16 and Figure 17). The presence of neural invasion (Figure 18) and emboli at the level of small vessels was highlighted (Figure 19 and Figure 20). The HP and IHC aspects indicate invasive carcinoma NST.

After surgery, the patient’s local evolution was a favorable one, and they started adjuvant chemotheraphy (Carboplatin + Gemcitabine) in November 2022 as well as radiotherapy (radiation dose of 50 Gy) between December 2022 and January 2023. From January 2023, the patient started treatment with 2.5 mg of Letrozole once a day, two 150 mg tablets of Abemaciclib a day and Zoladex once a month.

**Figure 21:**
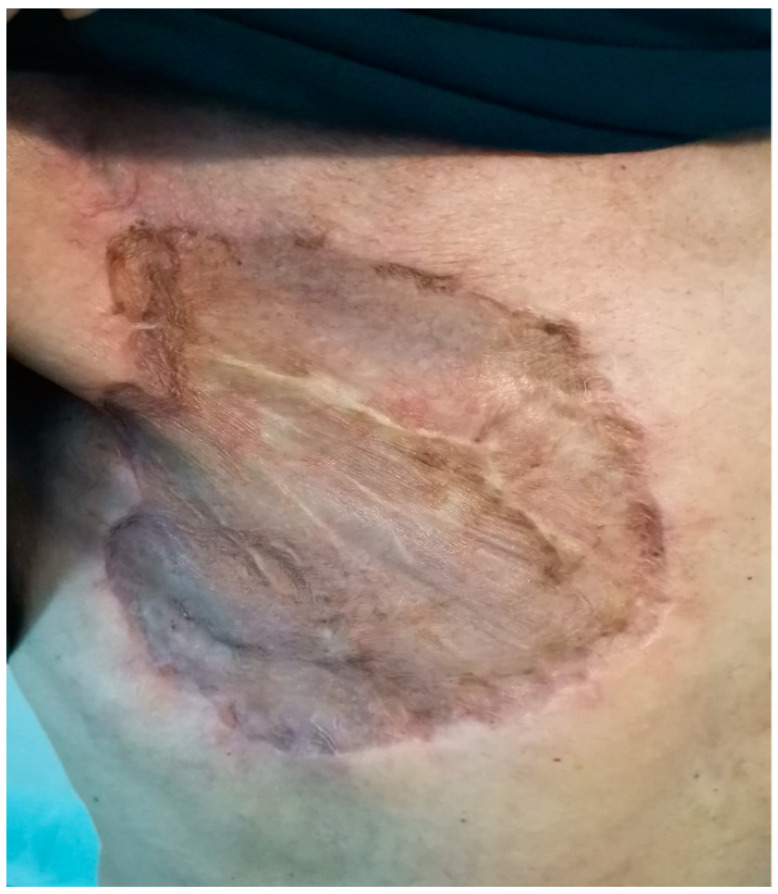
Appearance of the prepectoral area two months postoperatively.

**Figure 22:**
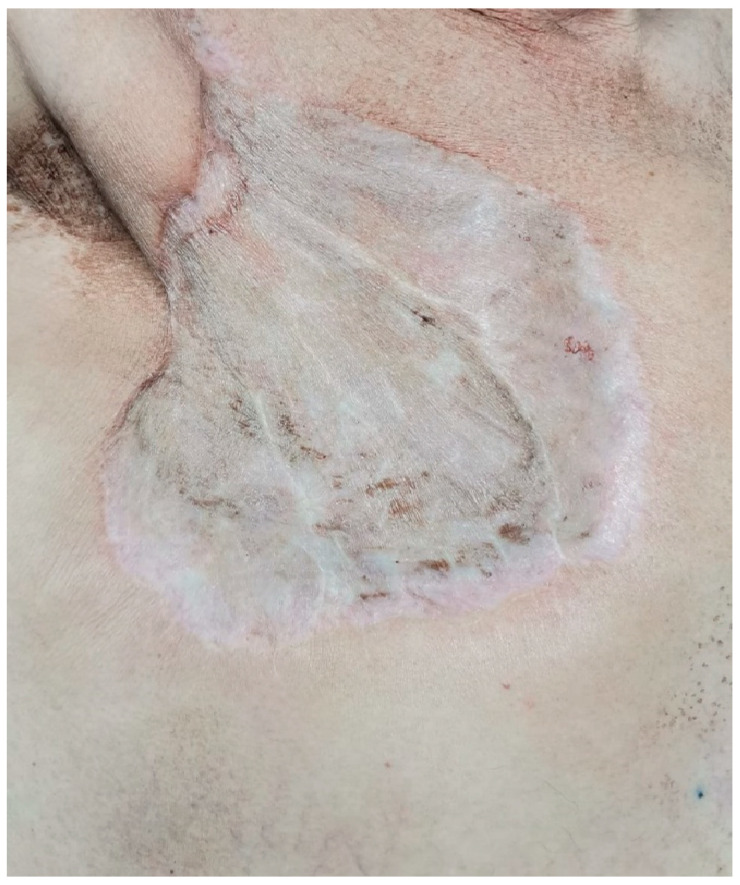
Appearance of the prepectoral area nine months postoperatively.

Surgical evaluation at 2 months (Figure 21) and 9 months (Figure 22) postoperatively showed favorable local evolution.

The CT scan performed 4 months and 9 months after the surgery showed the absence of suspicious lesions as secondary determinations or tumor recurrence.

## 3. Discussion

For a comprehensive view of MBC, we performed a literature review using PubMed and Google Scholar from 2014 to 2023 using the terms “male” and “breast cancer”; the filters “free full text” and “Aged: 65+ years” and the article type “case report”. We found 187 cases from which we excluded articles with insufficient information, cases of breast metastases from another type of cancer and articles that had other topics but had the keywords contained in them. We have extracted 16 cases of MBC, and these cases are described in Table 1 and Table 2 for further discussion.

In both men and women, BC incidence increases rapidly with age until the fifth decade of life. However, in men, the incidence continues to increase by the seventh decade, while women’s rate plateaus by the sixth decade [28]. Our patient was diagnosed at an advanced age. Another reason that explains the increase in the incidence in men is due to the lack of awareness about the existence of breast cancer in males.

Most of the cases in our review had as their main symptom the presence of a tumor mass in the breast or axilla. Eleven cases presented tumors larger than 2 cm in size [14,15,16,17,20,21,22,23,24,25,27], while four cases had smaller sizes [19,21,26].

The initial diagnosis of female BC often occurs at an earlier stage than in MBC, which is the reason why MBC frequently presents with more advanced features of the disease, correlated with lymph node involvement, larger tumor size, and metastases at the time of diagnosis [29,30]. Due to the low breast tissue in men, the tumor quickly invades the adjacent structures. Our case presented an advanced local invasion with a nodule at the level of the right mammary gland with a maximum diameter of 3 cm and multiple bilateral axillary, para-aortic and paratracheal adenopathies, without imagistic bone, lung or liver metastases.

Retroareolar lump is the most common clinical sign in MBC, which was the main and first symptom in our patient’s case as well, associated with axillary adenopathies, which, according to Cutuli et al., axillary nodal involvement is present in 50–60% of cases [31,32]. In contrast, our review detected the presence of axillary adenopathies in 5 out of 16 cases [14,15,16,26,27], respectively, in 31% of cases.

All patients underwent biopsy for diagnostic purposes, either with a core needle in five cases [14,15,16,18,22], fine-needle aspiration in four cases [23,24,25,27], or excisional biopsy in seven cases [17,19,20,21,26]. Given that MBC is rare and there are not enough surgeons specialized in breast surgery in our country, there is a lack of experience in the management and early diagnosis of this type of cancer. Our patient performed an incisional biopsy in a private clinic instead of a core-needle biopsy, which would have ensured an increased comfort of life and would have been diagnosed faster, and the oncological treatment would have started earlier.

The HP and IHC examinations have an extremely important role in the pathology of breast cancer [33]. Considering the very aggressive nature of the patient’s cancer with the presence of large tumors, multiple adenopathies, perineural invasion and emboli present in the vessels, the histopathological examination of the mastectomy piece suspected the micropapillary pattern, which was later disproved with the IHC examination. Epithelial membrane antigen (EMA) has a positive marking inside the cellular beaches (reversal of the polarity of the marking compared to an infiltrative micropapillary carcinoma that should have been peripheral to the cellular beaches), which helped us exclude the micropapillary pattern. EMA is also correlated with tumor size, tumor grade, progesterone, estrogen receptors and nodal stage [34].

Estrogen receptors are positive in 75–92% of the cases, while progesterone receptors are positive in 54–77%, according to Cutuli et al. [32,35]. Our case showed positivity for estrogen and progesterone receptors, as in the vast majority of MBCs. We have also noticed the presence of estrogen receptors (ER) and progesterone receptors (PR) in most cases from the literature review, with no HER2-positive case.

The expression of Ki67 is used as a proliferation marker, and it is an independent prognostic factor for survival rate [36]. A higher Ki-67 value (≥25%) was associated with a favorable response to chemotherapy, and his values significantly decreased to 10% after neoadjuvant chemotherapy [37,38]. E-cadherin is considered a tumor suppressor, and its loss has been demonstrated in invasive lobular carcinoma [39]. In our case, e-cadherin expression was maintained in the infiltrative areas, decreased in a solid area and preserved in another solid area, which demonstrated the heterogeneous character of the tumor. The IHC reactions performed on the biopsied breast tumor showed positivity for both types of receptors and HER2-negative, which are present in the vast majority of MBC [40,41].

Mastectomy combined with axillary lymphadenectomy for MBC is the surgical gold standard treatment and is more commonly performed (70% of all cases) in this type of cancer [42,43]. Surgery for locally advanced cancer should be performed in patients with a partial response to chemotherapy and who are hormone-positive to improve their quality of life [44]. In our case, mastectomy with axillary lymphadenectomy was performed for palliative purposes, but although the resection limits of the mastectomy piece were uninvaded and all 12 resected lymph nodes showed carcinoma metastasis, the response to adjuvant oncological treatment was favorable, with a control CT scan performed 4 months and 9 months after the surgery within normal limits. A favorable response was also observed with neoadjuvant chemotherapy, with a significant decrease in the Ki67 values from 56% to 10%. Neoadjuvant chemotherapy was received in 3 cases out of 16, respectively, in 18% of the cases from our literature review.

A study conducted among 411 people, men and women, at the Ankara Training And Research Hospital General Surgery Outpatient Clinic in 2021–2022 highlighted the low awareness of MBC in the general population. Only 38.9% of the study participants knew that men can also develop BC. The study noted that women have a higher level of BC awareness than men [45]. An important role in the early detection of MBC is played by education campaigns and the introduction of breast screening programs, at least for men with a family history of BC.

## 4. Conclusions

MBC is a rare and frequently neglected disease. It is important to understand the biological differences between male and female BC, which is why it is advisable to view them as two separate diseases. Given that MBC is rare, there is a lack of experience in the management and early diagnosis of this type of cancer, and there are also not enough methods of informing the population in our country about the existence of MBC. The treatment of MBC remains a challenge, and the development of therapeutic strategies is necessary. Also, there are not enough surgeons specialized in breast surgery and breast ultrasound in our country. Multidisciplinary collaboration and the early detection of people at risk and of the signs of BC are essential so that the diagnosis takes place as quickly as possible through the correct therapeutic method and sending the patient to a surgeon specialized in breast surgery. The HP and IHC examinations were essential in establishing the therapeutic attitude. Considering the fact that the patient presented at a very advanced stage (IIIC) of the disease, the treatment solution approached in our patient’s case led to very good results.

## Figures and Tables

**Table 1:** The main preoperative clinical characteristics across the literature reported cases of MBC between 2014 and 2023.

Study [Ref.]	Age (Years)	Side	Size [cm]	Clinical Characteristics	Axillary Lymph Node	Biopsy
Na Lin et al. [14]	78	Right breast	2.5 × 1.1 cm	No tenderness, no skin changes, no bloody fluid overflow at the nipple	Right axillary lymph nodes were palpable	Core needle biopsy—invasive ductal carcinoma
Basma Alsayed et al. [15]	82	Left axilla	8 × 7 cm	There were no masses felt in either breast	The overlying skin was erythematous, and there was a sinus discharging serous fluid.	Core-needle biopsy—invasive ductal carcinoma
Haruko Takuwa et al. [16]	69	Left breast	>6 cm	Tumor mass without skin invasion in the upper-lateral region as well as axillary lymph node swelling	Left axillary lymph node swelling	Core-needle biopsy—invasive ductal carcinoma
Bo Wang et al. [17]	73	Right breast	3.6 × 2.3 cm	Hard, smooth and movable lesion was palpated below the right papilla. There were no skin lesions	No palpable axillary lymph node	Breast tumor resection—a little papillary neoplasm of the breast with epithelial atypia and hypertrophy in the fibrous cystic wall with a little DCIS
Alain Mwamba Mukendi et al. [18]	68	Right breast	Not mentioned	Painless right breast lump	No palpable axillary lymph node	Core biopsy of the right breast—infiltrating ductal carcinoma displaying cribriform features.
Karan N Ramakrishna et al. [19]	69	Right breast	0.6 cm mass	Right-sided serous nipple discharge; nipple swelling and pain	No palpable axillary lymph node	Ultrasound-guided biopsy—atypical ductal hyperplasia Excisional biopsy—ductal carcinoma in situ
Chandler S Cortina et al. [20]	85	Left breast	2 cm	Pedunculated mass over the nipple and associated nipple and areola enlargement	No palpable lymph node	Excisional biopsy—invasive ductal carcinoma
Hua Luo et al. [21]	70	Left breast	2 cm	A well-circumscribed, firm and mobile mass in the left periareolar region.	No palpable lymph node	Excisional biopsy—intracystic papillary carcinoma
Hua Luo et al. [21]	67	Right breast	1.5	A well-circumscribed and firm mass in the right subareolar region. The tumor was fixed	No palpable lymph node	Excisional biopsy—intracystic papillary carcinoma
Hua Luo et al. [21]	76	Right breast	1 cm	Mobile lump in the right breast	No palpable lymph node	Lumpectomy—intracystic papillary carcinoma
Işıl Başara Akın et al. [22]	72	Right breast	4 cm	A painless, mobile lesion at the retroareolar region of the breast	No palpable lymph node	US-guided core needle biopsy—encapsulated solid papillary carcinoma
Swati Agrawal et al. [23]	65	Right breast	3 cm	Palpable mass in his right breast	No palpable lymph node	Fine-needle aspiration cytology—suggestive of malignancy
Swotantra Gautam et al. [24]	78	Left breast	3 × 2 cm	A non-tender lump just beneath the left nipple; it was mobile and not adhered to underlying structures	No palpable lymph node	Fine-needle cytology—carcinoma of breast
Mohammed Sekal et al. [25]	70	Left breast	4 cm	Nodule presented a rapid augmentation of its volume with adhesion to both superficial and deep plans and inflammatory opposite signs	No palpable lymph node	Fine-needle aspiration cytology—apocrine carcinoma
Manoj P Rai et al. [26]	81	Left breast	1.2 × 0.9 cm	Breast mass in the lower inner quadrant	Left axillary lymphadenopathy	Left breast lumpectomy—low-grade pleomorphic sarcoma
Soumya Sucharita et al. [27]	80	Right breast	6 cm	A well-circumscribed and firm mass. The corresponding skin surface was normal.	One right axillary lymph node was palpable	Fine-needle aspiration cytology was performed from both the breast mass and axillary lymph node—ductal carcinoma. Lymph node showed the features of reactive hyperplasia

**Table 2:** HP and IHC aspects across reported cases of MBC between 2014 and 2023.

Study [Ref.]	ER	PR	HER2	Ki 67	Neoadjuvant Chemotherapy	Surgical Treatment	Axillary Lymph Node Hp	Stage	HP of Specimen
[14]	90%	80%	Positive	70%	4 cycles: paclitaxel, capecitabine and trastuzumab	Modified radical mastectomy	reactive hyperplasia	T2N3M1	invasive ductal carcinoma
[15]	Negative	Negative	Negative	Not determined	No	Left modified radical mastectomy	4 out of 21 axillary lymph nodes showing metastatic disease	pT3N2M0	invasive ductal carcinoma
[16]	Positive	Negative	Negative	10%	No	Mastectomy and axillary dissection	36 out of 39 axillary lymph nodes showing metastatic disease	pT3N3aM0 (stage III)	invasive ductal carcinoma
[17]	Positive	Positive (20%)	Negative	20%	No	Breast tumor resection	no	Not mentioned	ductal carcinoma in situ
[18]	Positive (>91%)	Positive (70%)	Negative	30%	Tamoxifen 4 months	Right mastectomy and right axillary lymph node dissection	not mentioned	T4bN1Mx	invasive ductal carcinoma
[19]	Positive	Positive	Not mentioned	Not mentioned	No	Right total mastectomy + right axillary sentinel lymph node	no evidence of tumor spread to the lymph nodes	TisN0cM0	ductal carcinoma in situ
[20]	Positive	Positive	Equivocal	Not mentioned	No	Mastectomy with sentinel node biopsy	no evidence of tumor spread to the lymph nodes	T4bN0M0	invasive ductal carcinoma
[21]	Positive (90%)	Positive (>99%)	Negative	10%	No	Simple mastectomy with axillary sentinel lymph node biopsy	no positive axillary lymph node was detected.	Not mentioned	intracystic papillary carcinoma
[21]	Positive	Positive	Negative	35%	No	Mastectomy with sentinel lymph node mapping	no positive axillary lymph node was detected.	Not mentioned	intracystic papillary carcinoma with a small focus on invasive carcinoma
[21]	Positive	Positive	Negative	60%	No	Right mastectomy	not made	Not mentioned	intracystic papillary carcinoma
[22]	Positive	Positive	Negative	Not mentioned	No	Total mastectomy	not made	Not mentioned	encapsulated solid papillary carcinoma
[23]	Positive	Positive	Negative	Not mentioned	No	Right modified radical mastectomy with an axillary lymph node dissection	5 of 16 axillary nodes involved	T2pN1M0	invasive adenocarcinoma
[24]	Not mentioned	Not mentioned	Not mentioned	Not mentioned	No	Right modified radical mastectomy with an axillary lymph node dissection	no positive axillary lymph node was detected	T2N0M0	invasive breast carcinoma, NOS
[25]	Negative	Negative	Negative	Not mentioned	Palliative chemotherapy	No surgery	no surgery	Stage IV (lung metastases)	no surgery
[26]	Positive	Positive	Not mentioned	Not mentioned	No	No other surgery	no surgery	T1acN0M0	no surgery
[27]	Not determined	Not determined	Not determined	Not determined	No	Modified radical mastectomy	lymph node—reactive hyperplasia	Not mentioned	NOS type sarcoma

## Data Availability

The data presented in this study are available on request from the corresponding authors.
